# Effusive-Constrictive Pericarditis Associated With Parvovirus B19 Infection

**DOI:** 10.1016/j.jaccas.2024.102959

**Published:** 2025-01-22

**Authors:** Sebastian Hasslacher, Christopher Naoum, Malcolm Anastasius, Dianna Hanzek, Thomas Yeoh, Leonard Kritharides, John Yiannikas

**Affiliations:** a Department of Cardiology, Concord Repatriation and General Hospital, Hospital Road, Concord, New South Wales, Australia; b University of Sydney, Sydney, New South Wales, Australia; c Central Sydney Heart, Sydney, New South Wales, Australia

**Keywords:** CMR, constriction, echocardiography, parvovirus B19, pericarditis

## Abstract

Parvovirus B19, pathogen of children’s fifths disease, is among the causes of viral/idiopathic pericarditis. This paper presents 3 adult cases of complicated effusive-constrictive parvovirus B19 pericarditis presenting with overt diastolic heart failure during an outbreak in September to October 2023. Comprehensive transthoracic echocardiography and cardiac magnetic resonance played a key role in noninvasive assessment of constrictive physiology and pericardial inflammation, guiding successful treatment with low-dose prednisolone, having failed standard colchicine and nonsteroidal anti-inflammatory drugs.

## Graphical Abstract

**Figure:**
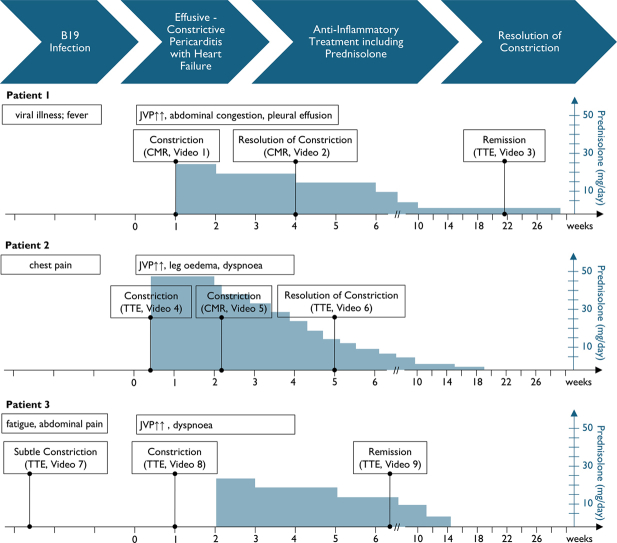

**Visual Summary:**
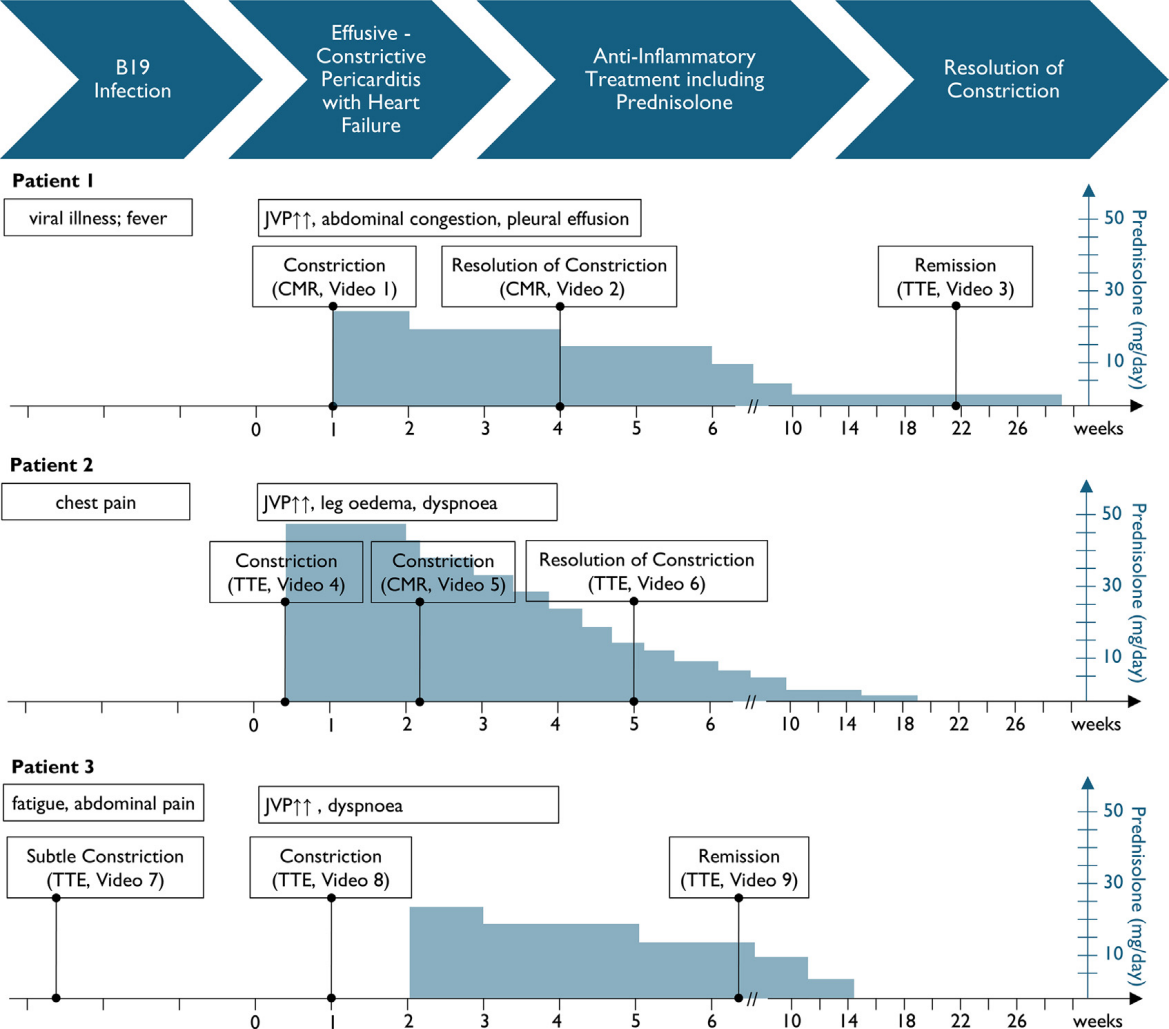
Clinical Events and Related Imaging Time course in weeks after hospitalization for heart failure due to effusive-constrictive pericarditis for patients 1 to 3. Symptoms and clinical findings before and at index hospitalization are indicated. Prednisolone doses (mg/d) and weaning regimen are displayed as blue area charts. Video sequences showing constrictive physiology at different stages are provided in the online appendix. CMR = cardiac magnetic resonance; JVP = jugular venous pressure; TTE = transthoracic echocardiography.

Pericarditis, defined by inflammation of the pericardial layers by various causes, is a common disorder accounting for approximately 5% of chest pain presentations to the emergency department.^1^ Although mostly self-limiting and benign, a minority of patients develop complications of pericarditis, including tamponade, chronic pericarditis, or constriction.^1^

**Take-Home Messages**

• Inflammatory effusive-constrictive pericarditis, a commonly underdiagnosed cause of heart failure, may rarely be caused by parvovirus B19 infection.
• Echocardiography and cardiac magnetic resonance play a key role in noninvasive assessment of constrictive physiology and allow effective treatment with anti-inflammatory therapy, which may prevent progression to chronic constriction.

Although parvovirus B19 infection is predominantly asymptomatic, it can sometimes be associated with a range of hematologic, dermatologic, rheumatologic, and pregnancy-related complications.^2^ The virus is spread by respiratory droplets, and epidemics typically occur during the spring in temperate climates. Although early viremia between days 4 and 8 is associated with fever and nonspecific influenza-like symptoms, rash and rheumatic symptoms can occur later due to antibody-related immune complex deposition.^2^ Erythroid progenitor are the natural host cells of parvovirus B19 in humans, and myocardial endothelial cells but not myocytes have been identified as parvovirus B19 target cells in the heart.^2^^,^^3^

We report a case series of 3 adults with complicated pericarditis during a parvovirus B19 outbreak between September and October 2023, in Sydney, Australia.

## Patient 1

**Figure 1:**
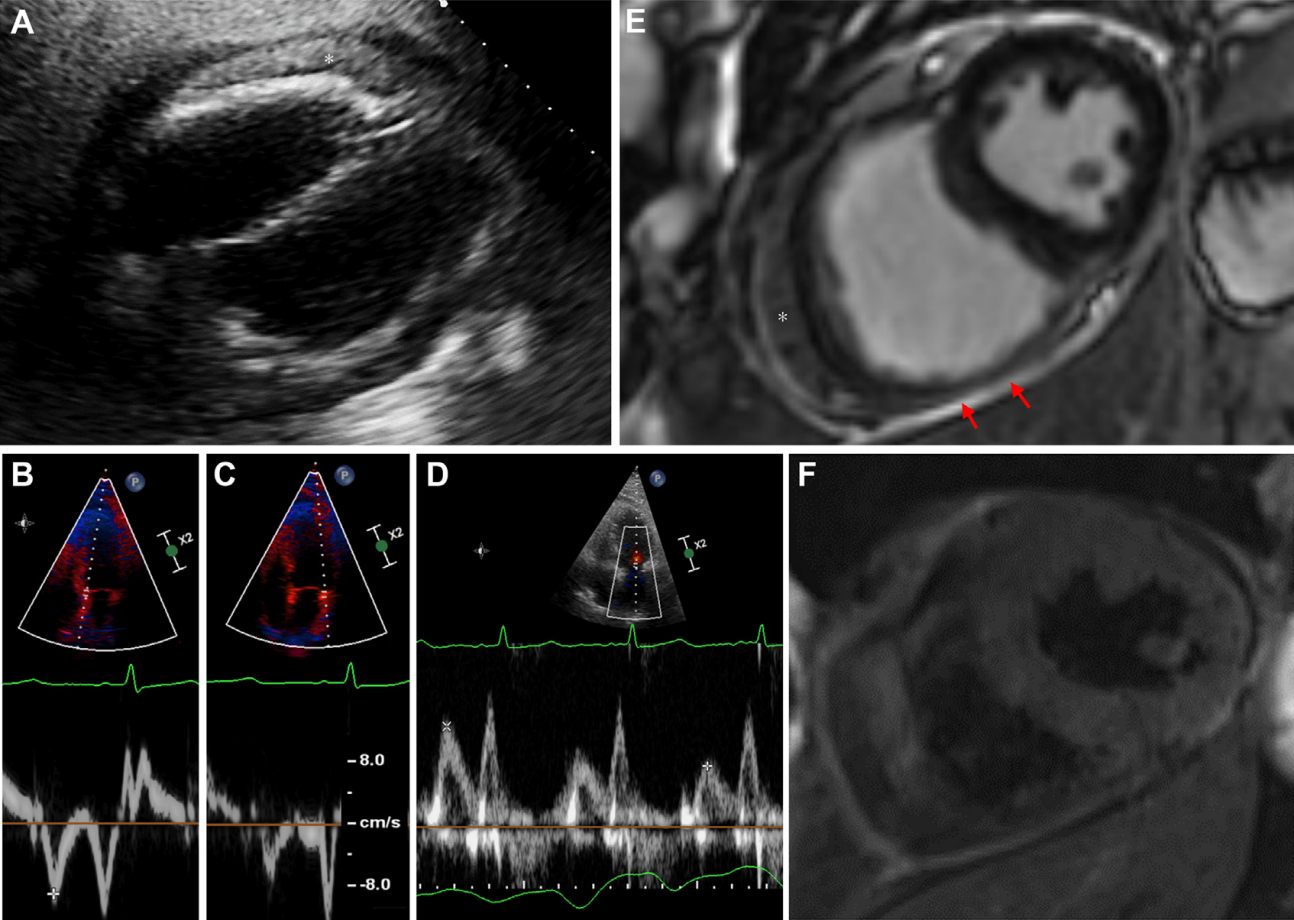
Imaging Findings of Patient 1 (A) Transthoracic echocardiography subcostal view displays moderate circumferential pericardial effusion without diastolic compression of right heart cardiac chambers. A prominent epicardial fat pad (∗) is noted adjacent to right ventricular free wall. (B) Septal mitral annular tissue Doppler velocity (e'_med_ = 9 cm/s) exceeding the (C) lateral mitral annular tissue Doppler velocity (e'_lat_ = 7 cm/s), demonstrating annulus reversus. (D) Pulsed wave Doppler shows significant respirophasic variation in mitral inflow velocity (E_exp_ = 69 cm/s, E_insp_ = 38 cm/s, percentage velocity change = 45%). (E) Cardiac magnetic resonance images 15 days after admission display pericardial thickening and delayed gadolinium enhancement on phase sensitive inversion recovery sequences (red arrows). (F) T2 black blood sequences demonstrate bright signal of the pericardial layers consistent with pericardial edema.

A 74-year-old man with a background of chronic coronary syndrome (CCS), hypertension, and type 2 diabetes presented to the emergency department with chest tightness, weight gain, and worsening dyspnea. He had recently returned from travel abroad where he had felt unwell with mild fever (38.5 °C), coryzal symptoms, and abdominal bloating. On initial assessment, the patient was hemodynamically stable but tachypneic and hypoxic with a respiratory rate of 26 breaths/min and a peripheral oxygen saturation of 84% on room air. Clinical examination revealed signs of biventricular congestive heart failure with severely elevated jugular venous pressure, mild-to-moderate bilateral pleural effusions, moderate ascites, and mild pitting edema to the ankles. A pericardial knock was not heard. C-reactive protein (CRP) was elevated at 147 mg/L, and liver enzymes were also mildly elevated in keeping with hepatic congestion. Echocardiography demonstrated a moderate size circumferential pericardial effusion (Figure 1A) with evidence of constrictive physiology (Video 1, Figures 1B to 1D) and normal biventricular systolic function. Troponin T levels were not elevated (6 ng/L, normal: <14 ng/L), excluding myocardial involvement. The patient was treated with intravenous diuretics and 500 μg colchicine twice daily for pericarditis. Cardiac magnetic resonance (CMR) demonstrated thickened pericardial layers with associated edema (increased T2 signal) and late gadolinium enhancement, consistent with acute pericarditis (Figures 1E and 1F). Parvovirus B19 immunoglobulin (Ig) M and IgG were positive, giving serologic evidence of recent parvovirus B19 infection, which was in keeping with the clinical history of infectious symptoms before hospitalization. Screening for other infectious, neoplastic, and autoimmune causes were noncontributory (Supplemental Tables 1 and 2). On day 6, treatment with 25 mg prednisolone was added due to persistent right heart failure and suspected effusive-constrictive pericardial disease. Subsequently, the patient’s clinical condition improved markedly with corresponding reduction in inflammatory markers. The patient was discharged after 19 days, with a prednisolone weaning regiment of 5 mg every fortnight. A follow-up CMR 2 weeks after discharge revealed near complete resolution of constrictive physiology (Video 2). The patient remained on 5 mg prednisolone due to a persistent mild interventricular septal motion abnormality seen on echocardiography at 5-month follow-up (Video 3) with no other features of constriction. Prednisolone was ceased 6 months after hospitalization, whereas colchicine was maintained.

## Patient 2

An 81-year-old man with a background of type 2 diabetes, hypertension, chronic kidney disease, and CCS presented to the emergency department with intermittent back and chest pain. On presentation, the patient was hemodynamically stable and clinical examination was unremarkable. Electrocardiogram (ECG) showed mild ST-segment elevation in leads V_2_ and V_3_ with right bundle branch block. Echocardiography showed normal biventricular systolic function without regional wall motion abnormalities with minimal pericardial effusion anterior to the right ventricle and adjacent to the inferolateral left ventricle. Computed tomography aortogram excluded acute aortic syndrome; serial troponin T levels were adynamic (15 and 15 ng/L, normal: <14 ng/L). A nuclear stress sestamibi scan was negative for inducible myocardial ischemia. Three days after admission, the patient developed a mild fever (38.1 °C), and biomarkers for inflammation were elevated with a CRP of 178 mg/L and white blood cell count of 11.7 × 10^9^/L. Urine culture showed mixed growth, and the patient was discharged on a course of antibiotics for presumed urinary tract infection.

**Figure 2:**
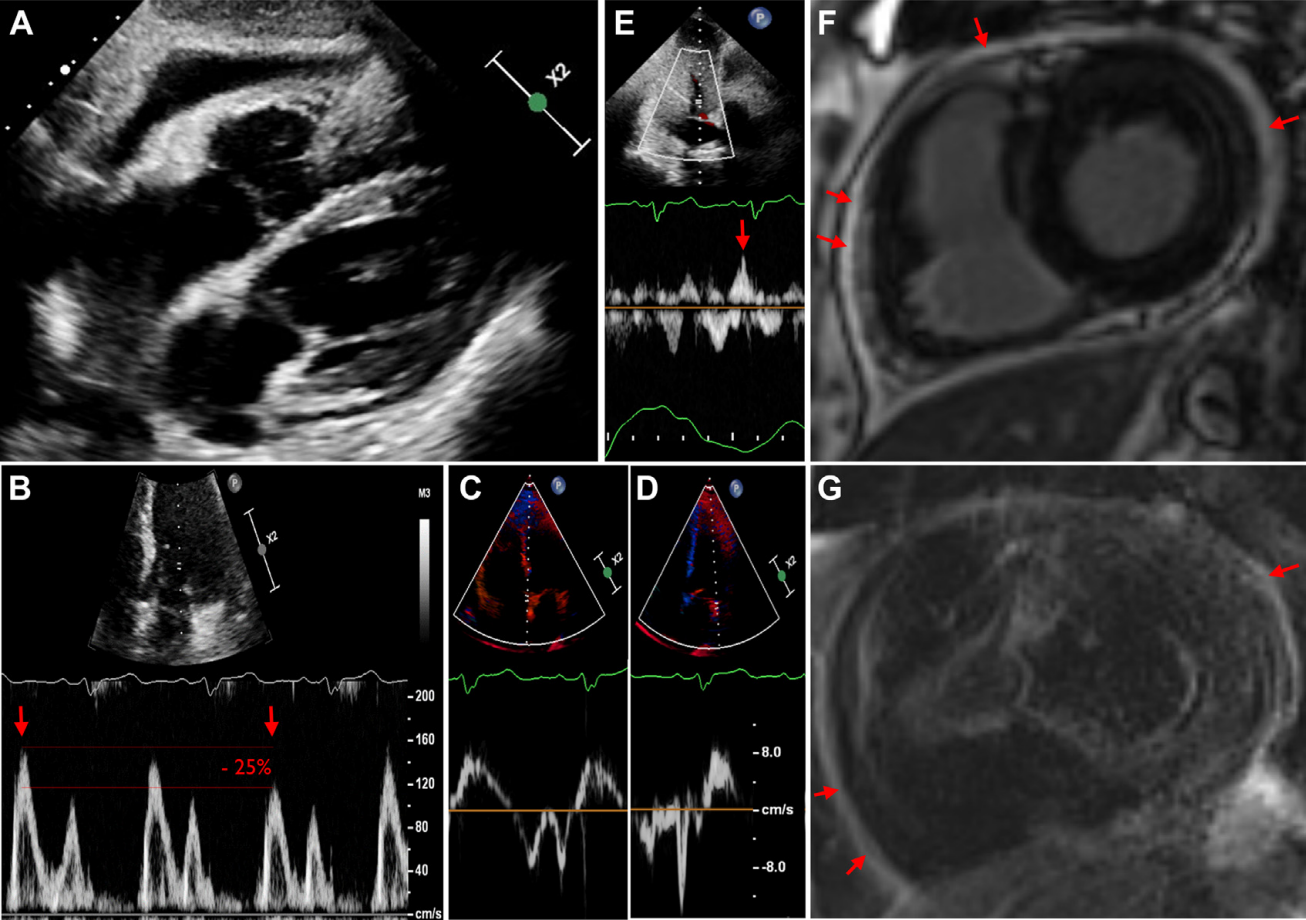
Imaging Findings of Patient 2 (A) Transthoracic echocardiography subcostal views show moderate circumferential pericardial effusion without diastolic compression of right heart chambers. (B) Pulsed wave Doppler demonstrates significant respiratory variation in mitral early diastolic inflow velocity (25% decrease with inspiration, red arrows). (C) Septal mitral annular tissue Doppler velocity (e'_med_ = 8 cm/s) exceeding the (D) lateral mitral annular tissue Doppler velocity (e'_lat_ = 6 cm/s), demonstrating annulus reversus. (E) Pulsed wave Doppler sampling of the hepatic veins demonstrating end-diastolic expiratory flow reversal (red arrow). (F) Cardiac magnetic resonance images 15 days after admission display pericardial thickening and delayed gadolinium enhancement in phase sensitive inversion recovery sequences (red arrows). (G) T2 black blood sequences demonstrate pericardial edema (red arrows).

Twelve days after discharge, the patient represented with episodes of sharp chest pain, dyspnea, and hypoxemia. Clinical findings were suggestive of predominantly right heart failure and pericarditis including markedly elevated jugular venous pressure, pitting edema to the knees, and a pericardial friction rub; however, a pericardial knock was not audible. ECG demonstrated intermittent atrial fibrillation with a heart rate between 78 and 140 beats/min. Blood tests revealed anemia (hemoglobin 83 g/L), acute on chronic renal failure (glomerular filtration rate 21 mL/min/1.73), elevated N-terminal pro–B-type natriuretic peptide of 3479 ng/L, and a mildly elevated troponin T level of 25 ng/L. Inflammatory biomarkers were elevated with a CRP of 107 mg/L, white blood cell count of 10.3 × 10^9^/L, and erythrocyte sedimentation rate of 85 mm/h. Echocardiography showed a moderate circumferential pericardial effusion (Figure 2A) with features of constrictive physiology including respirophasic interventricular septal shift (Video 4, Figures 2B to 2E). CMR on day 15 showed features of pericarditis and constriction including pericardial thickening (4 mm), pericardial edema with delayed enhancement, and respirophasic interventricular septal shift in free breathing cine sequences (Video 5, Figures 2F and 2G). The patient was treated with intravenous diuretics. Nonsteroidal anti-inflammatory drugs (NSAIDs) and colchicine were contraindicated due to acute on chronic renal failure (glomerular filtration rate 15 mL/min/1.73). IgM and IgG antibodies for parvovirus B19 were positive indicating recent infection. Other extensive etiologic investigations for pericarditis were negative (Supplemental Tables 1 and 2). Two days after admission, therapy with 50 mg prednisolone was commenced due to worsening heart failure due to pericardial constrictive physiology. Consequently, the patient’s clinical condition improved markedly and prednisolone was weaned from day 10 onward. Colchicine was subsequently commenced as kidney function recovered. Close clinical and echocardiographic outpatient follow-up revealed resolving pericardial constriction (Video 6). Prednisolone was weaned slowly by 5 mg every 5 days to a dose of 10 mg daily and then by 2.5 mg fortnightly thereafter and then ceased. Twenty-one weeks later, there was no evidence of recurrent pericarditis.

## Patient 3

**Figure 3:**
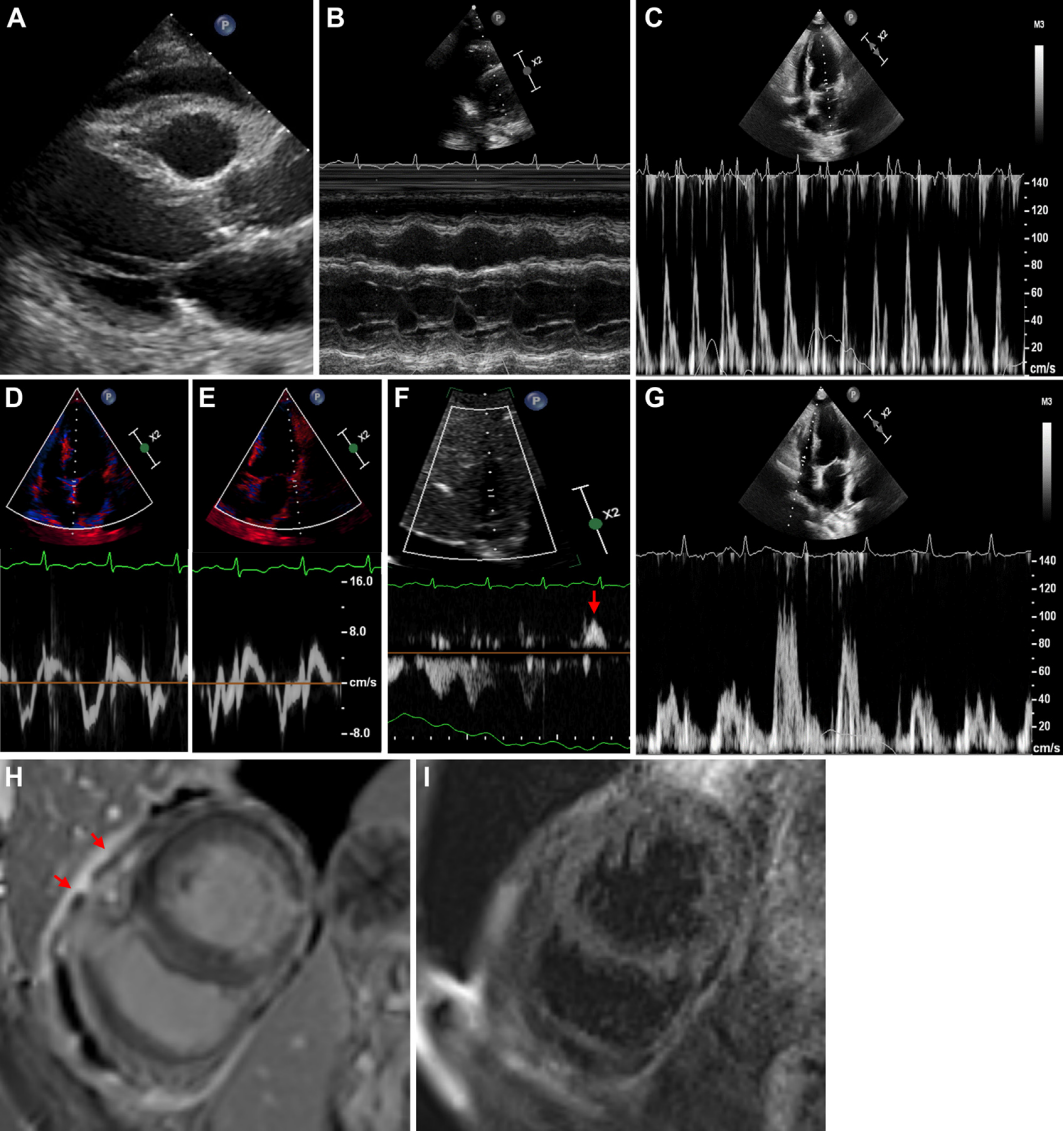
Imaging Findings of Patient 3 (A) Transthoracic echocardiography parasternal long-axis view displaying mild-to-moderate pericardial effusion adjacent to the right ventricular free wall at end diastole. (B) M mode of the parasternal short axis demonstrates significant respirophasic septal shift. (C) Mitral and tricuspid (G) inflow velocity demonstrates significant respiratory variation (41% and 59%, respectively). (D and E) Pulsed wave tissue Doppler of the mitral annulus medially (D) and laterally (E) both 8 cm/s. (F) Pulsed wave Doppler sampling of the hepatic veins demonstrating end-diastolic expiratory flow reversal (red arrow). (H) Cardiac magnetic resonance, 14 days after admission, displays pericardial thickening and delayed gadolinium enhancement on phase sensitive inversion recovery sequences (red arrows). (I) T2 black blood sequences demonstrate increased signal of the pericardial layers consistent with pericardial edema.

A 74-year-old woman with a background of chronic schizophrenia, CCS, and cardiovascular risk factors presented to the hospital with 3 days of fatigue and general decline. Initial investigations identified *Klebsiella pneumoniae* urosepsis and acute renal failure, and intensive care unit admission was required for vasopressor support. On day 2, echocardiography identified a mild-to-moderate pericardial effusion with subtle abnormal interventricular septal motion (Video 7). Concomitant pericarditis was diagnosed, and therapy with colchicine 500 mg once daily was started. The patient was discharged 13 days after admission. Eleven days after discharge, the patient represented with dyspnea. Physical examination showed severely elevated jugular venous pressure above the angle of mandible. A pericardial knock could not be heard. ECG showed sinus rhythm with T-wave inversion in the inferior and lateral leads. Serial troponin T levels were adynamic (20 and 20 ng/L, normal: <14 ng/L), excluding myocardial infarction or myocarditis. CRP was elevated at 151 mg/L. Echocardiography showed features of constrictive physiology including pronounced respirophasic interventricular septal motion (Video 8, Figures 3A to 3G). CMR confirmed acute pericarditis with mildly thickened (4 mm) and edematous pericardium (Figures 3H and 3I). IgG antibodies for parvovirus B19 were positive, and extensive screening for other infectious and immunologic causes was negative (Supplemental Tables 1 and 2). Colchicine dose was increased to 500 μg twice daily, and 25 mg prednisolone was commenced given extensive pericardial inflammation and constrictive physiology. The patient’s clinical status improved significantly over the following days and she was discharged 17 days later. One month after discharge, the patient was well without evidence of pericardial effusion or constrictive physiology (Video 9), and the prednisone dose was slowly weaned.

## Discussion

Although there are prior descriptions of parvovirus B19–related constrictive pericarditis requiring pericardiectomy in children,^4^^,^^5^ this is, to our knowledge, the first case series presenting parvovirus B19–related effusive-constrictive pericarditis in adults, presenting with heart failure and showing response to treatment with cardiac imaging–guided anti-inflammatory therapy.

This case series highlights the important aspects of clinical assessment and advanced cardiac imaging to diagnose constrictive pericarditis and guide the early implementation of immunosuppressive treatment in the setting of a unique etiology of parvovirus B19 pericarditis. First, differentials for pericarditis, including myocardial infarction and myocarditis, should be addressed, especially on the background of CCS. Parvovirus B19 has been linked to myocarditis and eventually dilated cardiomyopathy, and evaluation of myocardial involvement by assessing biomarkers for myocardial injury is recommended.^1^ These were negative in these patients.

Second, etiologic evaluation of pericarditis is challenging because direct pericardial histopathology is generally not available. Serum viral antibodies correlate poorly with cardiotropic viruses in pericardial tissue and fluid; therefore, routine viral serology is not recommended.^1^ However, comprehensive etiologic evaluation is reasonable in complicated pericarditis (eg, constriction) to identify treatable conditions (eg, HIV, tuberculosis, systemic inflammatory diseases) or latent infections (eg, hepatitis); these should also be identified because reactivation of infection may occur with immunosuppression for pericarditis. Although the exact mechanism of viral pericarditis—whether by direct viral replication or indirectly by autoimmune or autoinflammatory mechanism—can be difficult to establish, in our series, parvovirus B19 was the likely etiology for pericarditis in patients 1 and 2 given the preceding viral illness, positive parvovirus B19 serology (including IgM antibodies), and subsequent pericarditis. Although patient 3 was only positive for parvovirus IgG antibodies, the delayed diagnosis of pericarditis likely explains the absence of IgM seropositivity, given IgM antibodies are present only for 2 to 3 months after acute infection.^2^

Third, as shown in this case series, cardiac imaging is essential for the accurate identification of pericarditis and its associated complications (eg, pericardial constriction) and for guiding the use of immunosuppression based on the resolution of pericardial inflammation. To support a diagnosis of pericardial constriction, cardiac imaging is required to identify features of dissociation between intrathoracic and intracardiac pressures and exaggerated ventricular interdependence. Although comprehensive echocardiography can identify constrictive physiology, CMR is the first-line noninvasive modality for demonstrating active pericardial inflammation, and thus identifying patients likely to have reversible pericardial constriction with immunosuppressive therapy. Echocardiography is highly specific for the diagnosis of pericardial constriction (specificity 97%) if there is evidence of both respirophasic interventricular septal shift (Videos 1, 4, 5, and 8) combined with a high septal mitral annular velocity (e' > 8 cm/s) that exceeds the lateral mitral annular velocity (annulus reversus) (Figures 1B, 1C, 2C, and 2D), and a plethoric inferior vena cava and hepatic vein Doppler expiratory end-diastolic flow reversal (Figures 2E and 3F).^6^ The presence of a pericardial effusion with these features of constrictive physiology may be used for the noninvasive diagnosis of effusive-constructive pericarditis, as in the case of these patients.^1^ CMR pericardial late gadolinium enhancement sequences (Figures 1E, 2F, and 3H) correlate with histologic findings of neovascularization, and T2-weighted sequences (Figures 1F, 2G, and 3I) are sensitive for detection of relative increase in pericardial water content and thus inflammation.^7^ CMR may also demonstrate exaggerated ventricular interdependence in free-breathing cine images (Video 1) supporting pericardial constriction, which may be of use when echocardiographic image quality is limited.

Fourth, the integration of clinical assessment and cardiac imaging is necessary for the appropriate use of anti-inflammatories (eg, NSAIDs, colchicine), the mainstay of treatment for patients with acute and recurrent pericarditis, with colchicine known to significantly improve rates of remission and decrease the rate of pericarditis recurrence.^1^ Corticosteroids have been linked to increased risk of pericarditis recurrence and chronicity of illness and are thus generally used when there is a contraindication to use of NSAIDs/colchicine (eg, in patient 2 with renal failure), or lack of response to initial standard treatment.^1^^,^^8^ Although anti-interleukin-Iβ agents have recently been successful for constrictive pericarditis associated with incessant or recurrent pericarditis, we are unaware of its use in acute parvovirus-related pericardial disease.^9^ Patients in this case series demonstrated features of effusive-constrictive pericarditis and diastolic heart failure, requiring escalation of immunosuppression, given either lack of initial response to treatment or contraindication to NSAIDs/colchicine. Early anti-inflammatory treatment of pericardial constriction with CMR-proven pericardial inflammation may lead to reversal of constrictive physiology, effusion, and potentially avoid the need for cardiac surgery.^10^ After corticosteroid treatment, these patients demonstrated resolution of clinical heart failure and gradual reversal of pericardial constrictive physiology and inflammation on follow-up echocardiography and CMR, respectively, at 3- to 6-month intervals. Remission persisted after weaning of prednisone.

## Funding Support and Author Disclosures

Dr Kritharides has received grants from the NSW Government Department of Health, Heart Foundation of Australia Vanguard, and Medical Research Future Fund Australia; consulting fees from Commonwealth Serum Laboratories; and honoraria for lectures or educational work from Novartis and Amgen; and participates on the CHFlt Clinical Trial and ALDO BP Clinical Trial advisory board. All other authors have reported that they have no relationships relevant to the contents of this paper to disclose.

## Appendix

Video 1 Patient 1: Constriction Cardiac magnetic resonance free breathing short axis SSFP cine sequence demonstrating significant respirophasic septal motion

Video 2 Patient 1: Resolving Constriction Cardiac magnetic resonance free breathing short axis SSFP cine sequence demonstrating very mild respirophasic septal motion

Video 3 Patient 1: Remission Transthoracic echocardiography free breathing parasternal short axis sequence without respirophasic septal motion

Video 4 Patient 2: Constriction Transthoracic echocardiography free breathing parasternal short axis sequence demonstrating significant respirophasic septal motion

Video 5 Patient 2: Constriction Cardiac magnetic resonance free breathing short axis SSFP cine sequence demonstrating significant respirophasic septal motion

Video 6 Patient 2: Resolving Constriction Transthoracic echocardiography free breathing parasternal short axis sequence with mild respirophasic septal motion

Video 7 Patient 3: Subtle Constriction Transthoracic echocardiography free breathing parasternal short axis sequence with presumed respirophasic septal motion

Video 8 Patient 3: Constriction Transthoracic echocardiography free breathing parasternal short axis sequence demonstrating significant respirophasic septal motion

Video 9 Patient 3: Remission Transthoracic echocardiography free breathing parasternal long axis sequence without respirophasic septal motion

## References

[1] Adler Y., Charron P., Imazio M. (2015). 2015 ESC guidelines for the diagnosis and management of pericardial diseases. Eur Heart J.

[2] Young N.S., Brown K.E. (2004). Parvovirus B19. N Engl J Med.

[3] Pankuweit S., Klingel K. (2013). Viral myocarditis: from experimental models to molecular diagnosis in patients. Heart Fail Rev.

[4] Backhoff D., Steinmetz M., Ruschewski W., Stastny B., Kandolf R., Krause U. (2013). Severe constrictive pericarditis after parvovirus B19 and human herpes virus 6 infection in a 9-year-old girl. Pediatr Cardiol.

[5] Huang L., Zhou Q., Cui X. (2021). Diagnosis and etiological identification in severe constrictive pericarditis in a 14-year-old-girl: a case report. Transl Pediatr.

[6] Welch T.D., Ling L.H., Espinosa R.E. (2014). Echocardiographic diagnosis of constrictive pericarditis, Mayo Clinic criteria. Circ Cardiovasc Imaging.

[7] Chetrit M., Xu B., Kwon D.H. (2019). Imaging-guided therapies for pericardial diseases. JACC Cardiovasc Imaging.

[8] Kumar S., Khubber S., Reyaldeen R. (2022). Advances in imaging and targeted therapies for recurrent pericarditis. JAMA Cardiol.

[9] Andreis A., Imazio M., Giustetto C., Brucato A., Adler Y., De Ferrari G.M. (2020). Anakinra for constrictive pericarditis associated with incessant or recurrent pericarditis. Heart.

[10] Syed F.F., Schaff H.V., Oh J.K. (2014). Constrictive pericarditis – a curable diastolic heart failure. Nat Rev Cardiol.

