# Supplementary material for: Effusive-Constrictive Pericarditis Associated With Parvovirus B19 Infection
Source: JACC Case Rep. 2025 Jan 22;30(6):102959. doi: 10.1016/j.jaccas.2024.102959 (PMC12011158; doi:10.1016/j.jaccas.2024.102959)
Supplement: Supplemental Table 1 and 2 [file mmc10.docx]

**Supplemental Table 1 Infectious Etiology Work-Up for Patients During Index Hospitalization**

|  | Blood | | | | | | | | | | | | | | | | | | | | Nasopharynx | | | | |
| --- | --- | --- | --- | --- | --- | --- | --- | --- | --- | --- | --- | --- | --- | --- | --- | --- | --- | --- | --- | --- | --- | --- | --- | --- | --- |
|  | **Blood Culture** | **TB Gamma Interferon Assay** | **TB Antigen 1 (IU/mL)** | **TB Antigen 2 (IU/mL)** | **HIV (1+2) Antibody Status** | **Hepatitis B Surface Antigen** | **Hepatitis B Surface Antibodies (mlU/mL)** | **Hepatitis B Core Antibodies** | **Hepatitis B DNA Quantification (IU/mL)** | **Hepatitis C Antibodies** | **Hepatitis C Qualitative RNA** | **Hepatitis E Antibodies IgG** | **Hepatitis E Antibodies IgM** | **Human Herpesvirus 6 Antibodies IgG** | **Parvovirus B19 Antibodies IgM** | **Parvovirus B19 Antibodies IgG** | **Cytomegalovirus DNA** | **Cytomegalovirus Antibodies IgG** | **Cytomegalovirus Antibodies IgM** | **EBV Nuclear Antigen IgG** | **Respiratory Multiplex DNA/RNA^a^** | **Rapid SARS-CoV-2-RNA** | **Rapid Influenza A RNA** | **Rapid Influenza B RNA** | **Rapid RSV RNA** |
| Patient 1 | neg | neg | 0.03 | 0.07 | neg | neg | 2 | neg | n/a | neg | neg | neg | neg | neg | pos | pos | neg | n/a | n/a | pos | neg | neg | neg | neg | neg |
| Patient 2 | neg | neg | 0.00 | 0.00 | n/a | neg | 2 | pos | <10 | neg | n/a | n/a | n/a | n/a | pos | pos | n/a | pos | neg | pos | neg | neg | neg | neg | neg |
| Patient 3 | neg | ^b^ | 0.00 | 0.00 | neg | neg | 2 | neg | n/a | neg | n/a | n/a | n/a | n/a | neg | pos | n/a | n/a | n/a | n/a | n/a | neg | neg | neg | neg |

EBV = Epstein-Barr virus; Ig = immunoglobulin; n/a = not applicable; neg = negative test result; pos = positive test result; RSV = respiratory syncytial virus; TB = tuberculosis.

^a^Real-time polymerase chain reaction multiplex for respiratory DNA/RNA, including adenovirus, *Bordetella pertussis*, *Bordetella parapertussis*, *Bordetella* spp, human metapneumovirus, influenza A (subtypes H3N2 and H1N1), influenza 09, influenza B, parainfluenza 1 to 4, RSV, rhinovirus, enterovirus, parechovirus, *Mycoplasma pneumoniae*, *Chlamydophila psittaci*, *Legionella pneumophila*, *Legionella longbeachae*, *Legionella* spp, SARS-CoV-2, and seasonal coronavirus.

^b^Indeterminate because mitogen positive control did not respond adequately.

**Supplemental Table 2 Main Laboratory Parameters and Immunologic Work-Up During Index Hospitalization**

|  | **Age (years)** | **CRP (mg/L) peak** | **Troponin T (ng/L) peak** | **NTproBNP (ng/L) peak** | **Hb (g/L) nadir** | **Hb (g/L) baseline** | **Nuclear Antibodies Pattern** | **Nuclear Antibodies Titre** | **Extractable Nuclear Antigen Antibody^a^** | **Smooth Muscle Antibodies Titre** | **Smooth Muscle Abs Interpretation** | **Mitochondrial Antibodies Titre** | **Liver Kidney Microsomal Antibodies Titre** | **ANCA** | **ANCA Titer** | **Myeloperoxidase Antibodies** | **Proteinase 3 Antibodies** | **dsDNA Antibodies** | **Cyclic Citrullinated Peptide** | **Rheumatoid Factor** | **Immunoglobulin G Level (g/L)** | **Subclass IgG1 (g/L)** | **Subclass IgG2 (g/L)** | **Subclass IgG3 (g/L)** | **Subclass IgG4 (g/L)** |
| --- | --- | --- | --- | --- | --- | --- | --- | --- | --- | --- | --- | --- | --- | --- | --- | --- | --- | --- | --- | --- | --- | --- | --- | --- | --- |
| Patient 1 | 74 | 147 | 6 | 537 | 104 | 130 | ^b^ | 80 | neg | 20 | ^c^ | <20 | <20 | neg | <10 | n/a | n/a | neg | n/a | n/a | 13 | 7,5 | 3,9 | 0,3 | 0,4 |
| Patient 2 | 81 | 107 | 26 | 3479 | 82 | 111 | neg | <80 | neg | n/a | n/a | n/a | n/a | neg | <10 | n/a | n/a | neg | neg | n/a | 10 | 6,3 | 3 | 0,3 | 1,3 |
| Patient 3 | 74 | 151 | 33 | n/a | 83 | 108 | neg | <80 | neg | n/a | n/a | n/a | n/a | n/a | 10 | neg | neg | neg | neg | neg | 13 | 8,5 | 2,2 | 0,2 | 0,2 |

ANCA = anti-neutrophil cytoplasmatic antibody; CRP = C-reactive protein; Hb = hemoglobin; n/a = not applicable; neg = negative test result; NTproBNP = N-terminal pro–B-type natriuretic peptide; pos = positive test result.

^a^SS-A (Ro60), Ro-52, SS-B (La), RNP, Sm, Scl-70, Jo-1, proliferating cell nuclear antigen, PM-Scl, Ribosomal P, and centromere.

^b^Few nuclear dot, unknown significance.

^c^Pattern V, usually follows viral infections.
